# User experience and perceived usability of nurse-led telemonitoring among women with gestational diabetes in Dhulikhel, Nepal

**DOI:** 10.1007/s40200-024-01540-1

**Published:** 2024-12-16

**Authors:** Kalpana Chaudhary, Jyoti Nepal, Shraddha Thapaliya, Sangam Banjara, Abha Shrestha, Prabin Raj Shakya, Archana Shrestha, Shristi Rawal

**Affiliations:** 1 Department of Public Health, Kathmandu University School of Medical Sciences, Dhulikhel, Nepal; 2 Department of Research and Development, Dhulikhel Hospital, Kathmandu University Hospital, Dhulikhel, Nepal; 3 Department of Obstetrics and Gynecology, Dhulikhel Hospital, Kathmandu University Hospital, Dhulikhel, Nepal; 4 Biomedical Knowledge Engineering Lab, Seoul National University, Seoul, Korea; 5 Institute for Implementation Science and Health, Bhaktapur, Nepal; 6 Department of Chronic Disease Epidemiology, Yale School of Public Health, New Haven, USA; 7 Department of Clinical and Preventive Nutrition Sciences, School of Health Professions, Rutgers University, Newark, USA

**Keywords:** Pregnancy, Gestational diabetes mellitus, Telemonitoring

## Abstract

**Objective:**

To assess the usability and acceptability of nurse-led telemonitoring in managing gestational diabetes among Nepalese women.

**Methods:**

We conducted a convergent mixed-method study among 91 pregnant women diagnosed with gestational diabetes at Dhulikhel Hospital, Nepal. Participants received glucometers and blood pressure monitors, along with training and instructions to measure and record their blood pressure and glucose levels at home once a week. Starting from the 28th gestational week, the study nurse reviewed measurements obtained at home during the biweekly telemonitoring follow ups, alternating with hospital visits. We used the System Usability Scale (SUS) to assess perceived usability and conducted in-depth interviews to understand participants’ experiences with telemonitoring and related technologies, including feasibility, acceptability, satisfaction with treatment, usability, as well as any difficulties or unmet needs. The quantitative analysis included descriptive statistics to summarize participant characteristics and System Usability Scale (SUS) responses, while a framework analysis was applied to examine the qualitative data.

**Results:**

The mean SUS score for telemonitoring services was 72.1 ± 7.6, indicating good usability (a score ≥ 68 indicates good usability). 93% of participants wanted to use the service frequently; 88% found it easy to use; 81% considered it well-integrated with their typical prenatal care. Participants acknowledged the benefits of virtual health visits, such as frequent health monitoring, facilitation of communication with healthcare providers, appointment reminders, added motivation for home monitoring, increased access to health information, and prevention of unnecessary anxiety. Overall, participants expressed satisfaction with the quality and features of the nurse-led telemonitoring for managing gestational diabetes, emphasizing its role in ensuring uninterrupted prenatal care.

**Conclusions:**

Telemonitoring is a feasible and acceptable tool to facilitate close monitoring of pregnant women with gestational diabetes in peri-urban hospital settings in Nepal.

## Background

Gestational diabetes mellitus (GDM), a condition characterized by high blood sugar levels that develop during pregnancy, occurs in 2% and 16% of all pregnancies [1], with rates in Asia, ranging from 1.2 to 49.5% [2]. Nepal, a country in South Asia, has a reported GDM prevalence ranging from 4.81 to 28% [3–5]. Women with high-risk pregnancy complications such as GDM require frequent antenatal follow-ups throughout pregnancy. The cornerstone of GDM management is glycemic control by diet and lifestyle modifications and/or medication. Adequate glycemic control in patients with GDM is critical to prevent associated adverse outcomes such as preeclampsia, large for gestational age babies, birth injuries, labor complications, and cesarean delivery [6–10]. In addition to regular consultations on diet and lifestyle management, patients with GDM require close monitoring of their glucose levels and frequent modification of treatment plans [6]. In low- and lower-middle-income countries (LLMICs; countries with a gross national income per capita of $4,515 or less) [11] like Nepal, where home glucose monitoring is uncommon [12], frequent antenatal follow-ups are paramount for regular glucose testing and monitoring.

Telehealth, which refers to remote healthcare delivery via telecommunications technology [13], offers a unique tool to facilitate continuity in prenatal care. Several studies have evaluated telehealth solutions for women with GDM in high-income countries [14–20]. Evidence from several meta-analyses [1, 21, 22], supports that telehealth can improve patient satisfaction and reduce costs while achieving better or similar pregnancy outcomes compared to standard in-person clinic visits among women with GDM. Further, telehealth programs appear to have high satisfaction and acceptance among GDM patients and healthcare providers [20, 23–25]. However, the use of telehealth for GDM management has been largely unexplored in LLMIC settings, where the majority of GDM cases occur [26]. In a low-resource, primarily rural setting like Nepal, telehealth has tremendous potential to optimize antenatal care by mitigating several dominant healthcare challenges, such as shortage of healthcare professionals and resources, and transportation or time barriers to accessing adequate antenatal care [27]. 

Since the early 2000s, the Government of Nepal has initiated telehealth programs that provide specialist support to rural health facilities for diagnosis and treatment of diseases. In the maternal health sector, provider-facing mobile apps have been piloted to monitor and coordinate antenatal care. These interventions have had limited success and failed to gain traction beyond project periods [28], presumably due to usability and technical challenges [29]. However, with recent advancements and the wide adoption of communication and digital technologies, the potential for telehealth as a viable care option has grown in Nepal. Internet penetration is 62.9% of the total population, with most consumers using the internet on mobile devices. Nepal’s mobile service penetration rate is close to 100%, and one in two adults own a smartphone [30]. With the rapid expansion of mobile communications systems, increasing digital literacy, and the advent of low-cost bio-monitoring devices, the opportunity is ripe for effective telehealth programs that benefit GDM patients and providers in Nepal. Therefore, we piloted a nurse-led telemonitoring program to provide services to women with GDM in a large hospital in Dhulikhel, Nepal, with the objective of assessing its usability and acceptability among our target population.

## Methodology

### Study design

We conducted a convergent mixed-method study among pregnant women diagnosed with GDM at Dhulikhel Hospital, Nepal.

### Study participants

Our study population was Nepali pregnant women with GDM diagnosis. GDM was diagnosed by applying the Carpenter and Coustan criteria to the 100-g oral glucose tolerance test (OGTT) [31]. 

### Inclusion & exclusion criteria

Pregnant women who (i) received antenatal care at Dhulikhel Hospital, (ii) received a GDM diagnosis, (iii) owned an android smartphone, (iv) had internet connectivity at home, (v) could understand and read Nepali, (vi) were less than 28 gestational weeks into pregnancy, and (vii) were 18 years of age or older, were enrolled in the study. Patients with learning difficulties or vision/hearing impairments were excluded.

### Study site

This study was conducted at Dhulikhel Hospital, a community-based tertiary-level university hospital of Kathmandu University in Nepal. Located in Dhulikhel, 20 km from the nation’s capital (Kathmandu), Dhulikhel Hospital has a catchment population of 1.9 million people and delivers approximately 3,000–4,000 babies annually.

### Sample size determination

We conducted this study as part of an exploratory randomized controlled trial (RCT) aimed at testing a new mHealth platform (GDM-DH) that supports self-management and treatment (including telemonitoring) for GDM patients [32]. Treatment-related change in glycemic control was the primary outcome of the RCT, and the study was adequately powered to examine whether mean change in fasting and 2-hour blood glucose levels from enrolment to six weeks postpartum differed significantly by the treatment condition. We used G*Power 3.1.9.2 to estimate the sample size for a repeated-measures ANOVA (within-between interaction). Assuming a medium effect size of 0.25 (partial eta squared = 0.06), an alpha error level of 0.05, nonsphericity correction of 1, and a correlation of 0.5 for two groups with two repeated measurements, we calculated that a sample size of 34 would achieve 80% power. To account for possible attrition, and to assess the impact of the GDM-DH platform on treatment-related changes in adverse perinatal outcomes associated with GDM (e.g., birthweight, labor induction), we extended the sample size and eventually enrolled 91 women diagnosed with GDM between September 2021 and June 2024.

All women (*n* = 91) enrolled in the RCT received the nurse-led telemonitoring program, and completed the quantitative assessments, which are presented descriptively in the study. Of these, 9 women were purposefully selected for the qualitative assessments, which involved in-depth interviews. The sample size for the qualitative aim was chosen based on evidence that 9 participants are likely to achieve 91% code saturation, providing sufficient depth to capture key themes in the qualitative analysis [33]. 

### Ethical consideration

Rutgers University and Nepal Health Research Council’s ethical/institutional review board approved the study (Ref number: 735/2019). Trained research assistants thoroughly explained the study’s objectives, risks, and benefits, as well as the participants’ right to voluntary participation and the option to withdraw at any time. The participation was voluntary. We obtained written, informed consent from participants who agreed to participate in the study.

### Participant recruitment

**Fig. 1:**
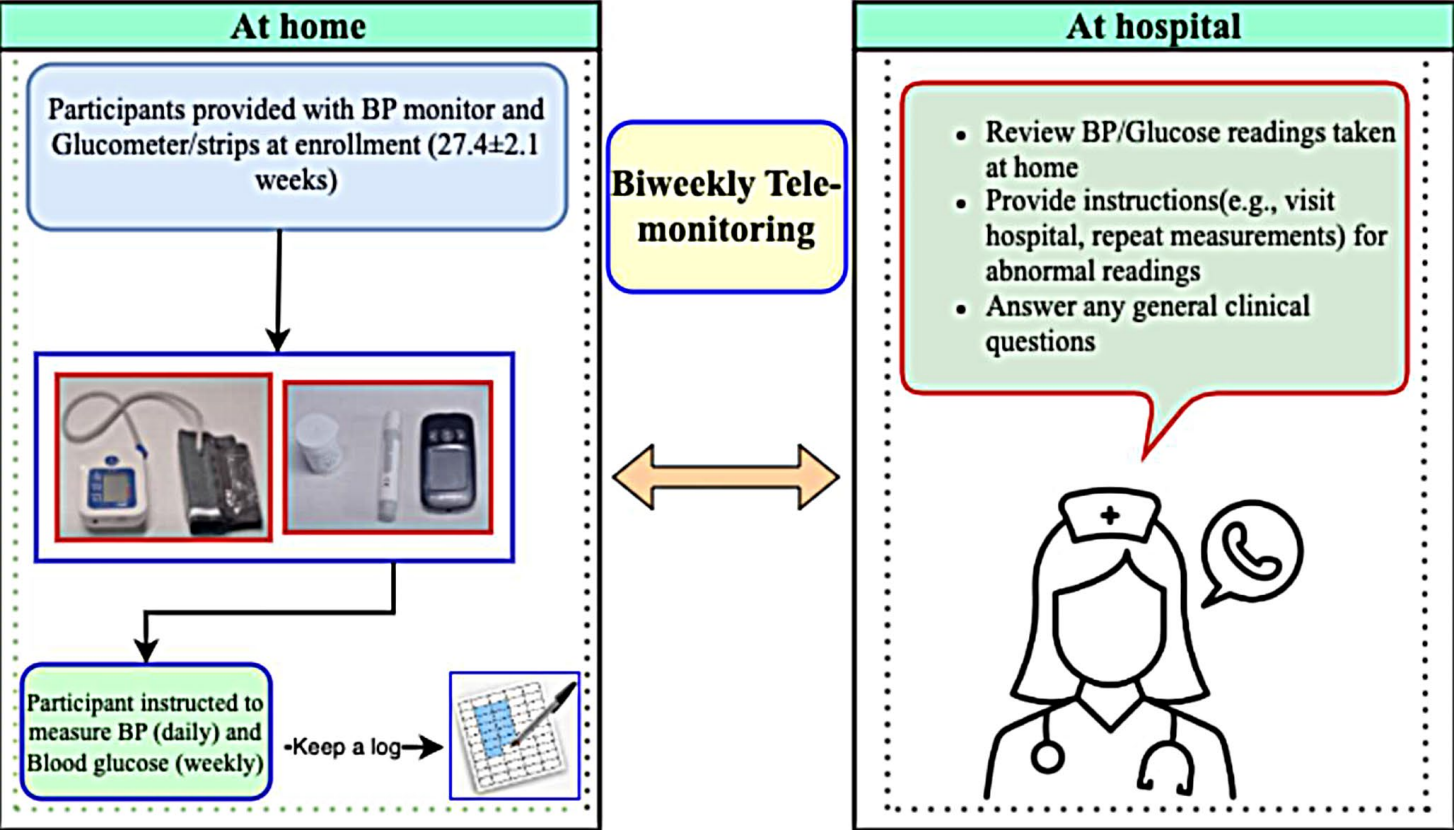
Overview of nurse-led telemonitoring program

All pregnant women receiving antenatal care at the Obstetric Outpatient Department (OPD) at Dhulikhel Hospital underwent routine screening for GDM at 24–28 weeks of gestation. If a patient received a GDM diagnosis, the OB-GYN physician or the hospital staff gave the patient some brief information about our study and/or referred them to the study coordinator with permission. The study coordinator contacted the potential study participant for screening, provided a detailed briefing of the study, and completed the consent procedures if the participant agreed to be in the study. A senior OB/GYN physician at the OPD directed all recruitment efforts with assistance from other OB/GYN physicians and hospital staff. Between September 2021 and June 2024, we enrolled 91 women at Dhulikhel Hospital who had received a GDM diagnosis during routine antenatal care and met the study’s other eligibility criteria (See Fig. 1).

### Nurse-led telemonitoring program

- Orientation to clinicians: Prior to the start of the study, all clinicians involved in providing care to GDM patients received a group orientation on the nurse-led telemonitoring program. Research staff assisted clinicians in setting up their unique login credentials and provided training on using the provider-facing web portal, enabling healthcare providers to access and manage patient data for treatment.
- Orientation to GDM patients and provision of glucometer and BP instrument: Each participant received a glucometer, a blood pressure (BP) monitor, and verbal instructions on their proper use for home monitoring. At enrolment, a study nurse trained the participants on how to measure and document blood glucose and BP at home. If an accompanying family member was present with the patient at their visit, they were also trained to assist with the measurements at home. Participants also received links to the video tutorials demonstrating the self-measurement process in the local language. Women could review these videos if they needed to revisit instructions regarding measuring their BP and blood glucose at home.
- Blood pressure and blood glucose measurements: Participants were asked to measure and record glucose values and BP at home at least once a week. The research team provided participants with enough glucometer strips to measure pre- and post-prandial glucose levels once a week, but they could buy their own strips to measure more than once a week. The participants were also asked to note the timing of food and beverage intake in relation to these measurements. Importantly, they were instructed to immediately contact the hospital hotline number for medical advice if their glucose or BP readings exceeded pre-specified threshold values (e.g., BP ≥ 140mmHg systolic or ≥ 90mmHg diastolic on two separate occasions) or if they experienced warning symptoms of hypoglycemia or preeclampsia, which were provided in a checklist beforehand.
- Bi-weekly telemonitoring follow up: During the biweekly telemonitoring follow up, the study nurse called each patient and reviewed the glucose and BP readings taken at home, along with any accompanying symptoms. The study nurse answered any of the patient’s queries and provided general clinical consultation. The study nurse also liaised between patients and other GDM service team numbers. If needed, follow-up calls with an OB/GYN physician and/or a dietician were scheduled to review the care plan and determine further management. Clinicians reviewed the data and suggested any needed changes to medications, diet, or treatment plans, or recommended hospital visits for further evaluation, if necessary. All women were instructed to keep their scheduled in-person appointments as per standard care.

### Quantitative data collection tool and variable measurement

Trained research assistants interviewed the participants in person and assessed socio-demographic characteristics including age in years, education, ethnicity, religion, marital status, employment and income (individual and household). We used the System Usability Scale (SUS) [33], a reliable and widely-used tool for global systems usability assessments containing 10 five-point Likert questions. In our main study, the internal consistency of the SUS responses achieved a Chronbach’s alpha of 0.79. The research team entered all data into a secure REDCap platform.

### Qualitative data collection tool and variable measurement

Of the participants enrolled in the study, a trained qualitative researcher interviewed 9 participants at 37–38 gestational weeks. All interviews took place in person in a research room at Dhulikhel Hospital, and the sessions were voice recorded with written consent from participants. We purposefully selected the participants for interviews based on differing compliance (high and low compliance) with the telemonitoring follow ups and demographic characteristics (age, ethnicity, occupation and education). The open-ended questions focused on gathering patient experiences with telemonitoring services and corresponding technology. Topics in the interview guide included feasibility, utility, acceptability (Please describe your overall experience with the care you received from our telemonitoring services for GDM management? ), satisfaction with care (What aspects of the telemonitoring service you received were most beneficial to you? ), challenges (Can you describe the challenges you have faced while using our telemonitoring services? ), and unmet needs (What suggestions do you have for improving the care we provide through telemonitoring service? ). Each interview lasted between 20 and 30 min.

### Quantitative data analysis

We presented quantitative data in frequencies and percentages for categorical variables, and as means and standard deviation for continuous variables. In the SUS, responses for individual questions range from 1 = Strongly disagree to 5 = Strongly agree. To compute the total SUS score for each respondent, we first converted the score for each question to a range from 0 to 4, with 4 being the most positive response. To do this, for all odd-numbered questions, which were positively worded, we subtracted 1 from the user response, and for even-numbered items, which were negatively worded, we subtracted the user response from 5. We then added up the converted responses for each user and multiplied that total by 2.5. This scaled the range of possible values from 0 to 100 instead of from 0 to 40, with each question weighing 10 points. A SUS score of 68 or higher indicated good usability [33]. We used SPSS 28 for all statistical analyses.

### Qualitative data analysis

We transcribed interviews verbatim and analyzed them using both deductive and inductive approaches through framework analysis [34]. Research assistants who were not present during the interviews transcribed the recordings into Nepali. To ensure accuracy, the interviewer compared these transcripts with the original audio recordings to identify and correct any discrepancies or omissions. The research team read the transcripts multiple times to become thoroughly familiar with the data. We employed the framework method to analyze the data and uncover themes related to telemonitoring services, including feasibility, satisfaction with care, challenges, and unmet needs. We started with predefined codes based on our existing knowledge and incorporated new codes as they emerged from the interviews. To identify meaningful units in this study, we followed a systematic and iterative approach. We first segmented the text into discrete sections that captured distinct ideas or concepts relevant to our study’s objectives. Each section was then coded both deductively and inductively to reflect participants’ perspectives and experiences. The interviewer developed a codebook by revisiting the transcripts to identify and define key themes. Codes that did not fit the existing themes were reorganized or grouped into new themes. To ensure that quotes and phrases were accurately aligned with the codes, we used a structured process. We carefully examined the text to select relevant quotes and mapped each to specific codes in the codebook. We compared these quotes with the code definitions and criteria to ensure proper alignment. This involved categorizing quotes based on their fit with thematic categories and codes. We iteratively refined the codes and themes as needed to accurately capture the data’s meaning. Regular reviews and discussions among the research team ensured that quotes were consistently and accurately aligned with the codes, maintaining the integrity of the coding process and ensuring that the final themes accurately represented participants’ views. During coding, notes were made to assist with interpretation. A second analyst applied the codebook to code the transcripts, and any discrepancies between the codes were resolved through discussion to reach consensus. After coding we used a spreadsheet to create a matrix and the data were charted into the matrix to draw inferences.

### Mixed methods integration

To determine whether the qualitative results supported or expanded our understanding of the SUS scores, we employed merging integration [35], which involved comparing qualitative findings with respect to SUS scores.

## Result

Table 1 describes the socio-demographic characteristics of the 91 participants included in the study. The mean age was 30.1 (SD = 4.4) years. The average years of schooling was 13.4 (SD = 3.2) years. Almost half of the participants belonged to the Brahmin/Chhetri (41.7%) and Newar (40.6%) ethnic groups. The majority of women followed Hindu religion (86.8%). All but 1 woman was currently married. About half of the participants were housewives or unemployed (52.7%). The median annual income of women was 1818 (IQR = 909.6) dollars, while the median household income was 4545.5 (IQR = 4545.6) dollars (See Table 2).

**Table 1:** Socio-demographic characteristics of the study participants, *n* = 91

Characteristics		All participants, *n* = 91	Qualitative, *n* = 9	*P*-value
		*n* (%)	*n* (%)	
Age in years (mean ± SD)		30.1 ± 4.4	29.6 ± 3.7	0.90
Education in schooling years (mean ± SD)		13.4 ± 3.2	14 ± 2.9	0.94
Ethnicity				0.83
	Brahmin/Chhetri	38 (41.7)	5 (55.6)	
	Newar	37 (40.6)	3 (33.3)	
	Magar/Tamang/Rai/Limbu	7 (7.6)	1 (11.1)	
	Kami/Damai/Sarki	4 (4.4)	0 (0.0)	
	Others	5 (5.4)	0 (0.0)	
Religion				1.0
	Hindu	79 (86.8)	8 (88.9)	
	Non-Hindu	12 (13.1)	1 (11.1)	
Marital status				1.0
	Married	90 ((98.9)	9 (100)	
	Separated	1 (1.1)	0 (0.0)	
Employment				1.0
	Service	24 (26.3)	2 (22.2)	
	Business	18 (19.7)	3 (33.3)	
	Home-maker	48 (52.7)	4 (44.5)	
	Farming	91 (1.1)	0 (0.0)	
Annual Income in $				
	Personal Income, Median (IQR)	1818.1 ± 909.1	2272.8 ± 727.2	0.93
	Annual per capita household income, Median (IQR)	4545.4 ± 4545.6	5454.5 ± 3030.3	0.82

^1 $ =132 rupees in Nepalese currency^

**Table 2:** System usability score for telemonitoring, *n* = 91

SUS scoring	*N* (%)
Poor score	22 (24.2)
Good score	69 (75.8)

^Poor score=0–68, Good score=69–100^

In the qualitative interview group (*n* = 9), the mean age of participants was 29.6 (SD = 3.7) years with an average of 14 (SD = 2.9) years of schooling. More than half (55.6%) of the participants were Brahmin/Chhetri. The majority of the participants (88.9%) were Hindus, and all the participants were married. The median annual income of women was 2272.8 (IQR = 727.2) dollars, while the median household income was 5454.5 (IQR = 3030.3) dollars. There were no statistically significant differences in sociodemographic characteristics between the quantitative and qualitative group of participants.

### Usability ratings

Overall, participants reported good usability of the telemonitoring services in managing GDM with a mean SUS score of 72.1 ± 7.6 (total possible score 100). About 75.8% of the participants gave good usability scores (> 68) for the telemonitoring service.

About 93% of participants expressed a desire to use this service frequently. Additionally, 88% of the participants found the service easy to use, while 81% considered it seamless and well-integrated into their regular health care plan. About 89% of the participants reported confidence in using the service. Less than 5% of the respondents felt that the telemonitoring services were unnecessary and complex (3.3%), in need of technical support (3.3%), or inconsistent (2.2%) (Table 3).

**Table 3:** Usability Assessment of the Telemonitoring Platform via the System Usability Scale, *n* = 91

System Usability Scale	Mean score	Positive response, *n* (%)
	µ ± SD	
I think that I would like to use this service frequently.	4.0 ± 0.4	85 (93.4)
I found the service unnecessarily complex	1.9 ± 0.6	3 (3.3)
I thought the service was easy to use	4.0 ± 0.4	80 (87.9)
I think that I would need the support of a technical person to be able to use this service	2.0 ± 0.5	3 (3.3)
I found the various function in this service were well integrated	3.7 ± 0.6	74 (81.3)
I thought there was too much inconsistency in this service	2.0 ± 0.5	2 (2.2)
I would imagine most people would learn to use this service very quickly	3.8 ± 0.5	73 (80.2)
I found the service very cumbersome to use	1.9 ± 0.5	15 (16.4)
I felt very confident using the service	3.9 ± 0.5	91 (89.0)
I needed to learn a lot of things before I could get going with this service	2.2 ± 0.8	11 (12.0)

^a^Scores for individual questions range from 1 = Strongly disagree to 5 = Strongly agree. To compute the total SUS score for each respondent, we first converted the score for each question to a range from 0 to 4, with scores of 3 and 4 considered positive responses, and 4 being the most positive response. To do this, for all odd-numbered questions, which were positively worded, we subtracted 1 from the user response, and for even-numbered items, which were negatively worded, we subtracted the user response from 5. We then added up the converted responses for each user and multiplied that total by 2.5. This scaled the range of possible values from 0 to 100 instead of from 0 to 40, with each question weighing 10 points

### Qualitative

We explored the participants’ experiences using the telemonitoring services under 4 themes: satisfaction, difficulty, service quality, and advice on improving the service.

### Satisfaction

Participants had an overall positive experience with the telemonitoring service. They were satisfied with it for several reasons.

The participants believed that the telemonitoring service was simple to use. They did not experience any difficulty in accessing the service. “*Telemonitoring service is useful. Everybody understands. It is helpful for everyone.” (P2)*.

Participants appreciated the continuous health monitoring and updates, which provided them with reassurance and a sense of safety. For instance, a 28-year-old woman stated that the telemonitoring service regularly inquired about her health and provided updates about her current health condition, which significantly reassured her about her health status, and prevented any unnecessary anxiety. “*Benefits of telemonitoring include being constantly watched after. This has made me more at ease knowing that my little one and I are secure.” (P7)* Another 31-year-old participant stated, *“They do a health checkup once a month in the hospital*,* but the nurse calls us in 2 weeks and informs us for blood sugar checkup. As they inform us of blood sugar checkups*,* I feel motivated to do so. I feel that if I do not do checkups on time*,* adverse consequences may arise. That’s why I feel happy when they call me.” (P4)*.

The participants expressed satisfaction with the program’s reminders for their medical appointments and blood glucose checks, as these provided added motivation for home monitoring and helped them stay on top of their health management more effectively. These calls provided an opportunity for the participants to discuss and address their health concerns without the need for in-person consultations at the hospital. They viewed the telemonitoring services as a valuable tool for prompting further consultation with their doctors. A 31-year-old woman expressed “*The day before yesterday*,* I forgot to check my blood glucose. When they called me*,* it clicked in my mind that I had forgotten to check and paid attention for checkup.” (P8)* She further added that telemonitoring services were similar to the services provided in person at the hospital.

Additionally, the personalized support and nutrition counseling received through the program made participants feel informed, motivated, and well cared for. A 34-year woman expressed. “*I feel very happy. I got the opportunity to learn about health and diseases. Not everyone gets this kind of opportunity to know if their sugar level has increased or decreased; if the disease has worsened*,* they don’t know. There is someone to rely on to clarify our health concerns*,* I feel very happy.” (P2)* The same women also added that telehealth visits facilitated communication with health providers and allowed them to clarify their doubts and queries. *“I feel happy when a study nurse calls me. Through telephone calls I can talk about my current health status and ask them about any concerns and queries regarding my health.” (P2)*.

Furthermore, the participants emphasized that telemonitoring services were crucial in overcoming the logistical barriers preventing access to care. For instance, a 28-year woman from a rural area who could not make regular physical consultations with the health provider due to financial and transportation constraints stated, *“Through telemonitoring services my health concerns reached the doctors.” (P7)*.

### Service quality

Participants were satisfied with the quality of the telehealth services, highlighting their ease of use and utility as a valuable source of health information. They perceived telemonitoring as a helpful intermediary between themselves and their healthcare providers. The regular monitoring of blood glucose levels was particularly noted for helping avert potential adverse outcomes. Overall, participants reported the quality of the telemonitoring service to be satisfactory. *“I am very happy*,* very nice service” (P9)*.

Participants felt that all aspects of their health were thoroughly addressed during the telephone consultations. The telemonitoring service effectively answered their health queries and allowed regular feedback on their blood glucose and blood pressure readings. They praised the quality of the service and recommended that the service be extended to all pregnant women, irrespective of their GDM status. One specific suggestion was to increase the frequency of the consultation phone calls to weekly instead of biweekly, particularly on the day before the glucose monitoring. One 28-year-old participant expressed *“This service is focused on women with high blood glucose. I think it would be very useful if the service is provided to all women.” (P7) Another 25 year old woman said*,* “Instead of two weeks this service should be provided weekly*,* since we check our blood sugar on Friday then if they call on Thursday we know beforehand…eh I got a call from hospital….I remember to check.”*

Overall, the framework thematic integration of SUS reports and open-ended interview participant responses revealed a good alignment between qualitative and quantitative data, enhancing our comprehension of the findings. Qualitative insights from the interviews provided detailed context and corroborated the accuracy of the survey responses. The mixed-method approach demonstrated that telemonitoring is a feasible and acceptable tool to facilitate close monitoring of women with GDM.

## Discussion

Women with GDM attending the outpatient clinic of a tertiary hospital in central Nepal rated the bi-weekly nurse-led telemonitoring program positively for usability, with the majority finding it easy to use and well-integrated into their healthcare routine. Most desired to use the service frequently, indicating its perceived value and convenience. Qualitative findings further underscored participant satisfaction, citing benefits such as regular health monitoring, feeling of safety, and access to reliable health information at home.

Participants in this study expressed overall satisfaction with the bi-weekly telemonitoring program, noting it was easy to use, and an overwhelming percentage of participants (93%) were willing to use the services again in future pregnancies. These findings are consistent with prior research indicating strong acceptance of telehealth interventions for managing high-risk perinatal conditions like GDM [25, 34–37]. Similar to our study findings, patients in other settings were inclined to use telemonitoring services due to the perceived support provided through telehealth [16, 36]. Our study also reinforces the technology acceptance model, highlighting that perceiving the technology as easy to use and beneficial is key to its sustained usage [38]. 

In the qualitative findings of this study, participants attributed their satisfaction with telemonitoring services to several factors, including reminders for health check-ups, increased motivation for home monitoring, access to health information, reduced travel time and costs for clinic visits, frequent health monitoring, improved communication between participants and healthcare providers, and alleviation of unnecessary anxiety. The perceptions and benefits of the telemonitoring services reported by patients in our study align with those reported in prior studies, where telemonitoring has been shown to enhance patient satisfaction by facilitating healthcare access [36, 37, 39], reducing the need for in-person clinic visits [15], and improving information exchange between patients and providers [40]. Similarly, in a qualitative study conducted in Ireland that examined the perception of mobile health (mHealth) solutions for diabetes during pregnancy, most women expressed a willingness to manage their condition from home while being remotely monitored by healthcare teams [41]. By making it easier for the patient to communicate their blood glucose values to the healthcare providers, and receiving feedback through phone consultations, patients might be able to better control their blood glucose levels through diet or physical activity adjustment [39]. This approach has significant potential to improve clinical outcomes and enhance the quality of life in patients with GDM [35]. 

The primary feature of the telemonitoring services that significantly boosted participants’ self-awareness and motivation was the ability to self-monitor blood glucose values, review their home-recorded blood pressure and glucose readings, receive guidance on abnormal readings, and have their health-related questions answered by a study nurse. Participants also recommended that the service be extended to all pregnant women irrespective of their GDM status so that they too could benefit from telemonitoring. In our study, participants particularly valued the nurse-led telemonitoring for its convenience, as well as the reminders for medical appointments and blood glucose checks, which helped them effectively stay on top of their health management. Other studies have similarly shown that telemonitoring services, including nurse follow-up for GDM and diabetes management, results in better glycemic control [18, 42], more frequent glucose monitoring, foot inspections, and weight monitoring, as well as fewer problems with medication adherence [43]. However, telehealth support for GDM management did not significantly impact service utilization or costs. Telehealth intervention resulted in comparable clinical outcomes to usual care, without compromising the quality of clinical care [18]. Telemonitoring enhances and strengthens the healthcare system by offering continuous care tailored to patient needs [43]. The ease of use of telemonitoring system plays a pivotal role in promoting their adoption, while systems that introduce additional workload amidst competing priorities can hinder their uptake and utilization [44, 45]. Overall, our study highlights the potential advantages of telemonitoring in enhancing patient self-management while preserving the quality of clinical care. It also underscores the critical importance of user-friendly systems and the role of telemonitoring as a valuable supplement within the healthcare continuum.

Our study has several strengths. First, this is the first study in Nepal to assess the perspectives and user experiences of telemonitoring among pregnant women with GDM. Second, participants had access to video tutorials for self-measurement of glucose and BP at home, in addition to having being trained in person by a study nurse during their antenatal visit. Third, the study employed a robust and rigorous mixed-method design to ensure comprehensive data collection and analysis. While we did not pre-specify a structured framework such as the four dimensions criteria [46], to ensure the rigor of our study, we employed several strategies that align with this established criteria for qualitative research. With respect to credibility, we pilot-tested the interview guide with 2 participants and had it reviewed by subject matter experts within our team. We also held team discussions to ensure consistent interpretation of themes and codes. Dependability was addressed by establishing intercoder reliability and adhering to a detailed study protocol. We ensured that the two coders involved in the study consistently interpreted the data and followed a comprehensive protocol throughout the study. To enhance confirmability, we used triangulation by integrating both qualitative and quantitative data, which helped validate our findings. Finally, we ensured transferability by reaching data saturation, making our findings robust and potentially applicable to other contexts or settings. The mixed-method design provided a more comprehensive understanding of the perspectives and experiences related to telemonitoring in GDM management. Triangulation of quantitative and qualitative data further strengthened the validity and reliability of our study. However, our study has several limitations. First, our study was intended as a feasibility study, and thus our sample size was limited. As a result, our findings cannot be generalized, and more studies with larger sample sizes are warranted. Second, the study only included literate women aged 18 and older who owned Android phones, limiting our ability to generalize the findings to other populations. Third, while we used the SUS to measure usability and feasibility, this tool, though validated in different countries, has not been validated in Nepal. However, we took several steps to address this limitation. We translated the SUS questionnaire into Nepali and back translated into English. We also conducted cognitive testing of the intended use of the tool in the pilot testing phase to address this limitation. Fourth, we did not assess the cost-effectiveness of the telemonitoring service. Lastly, the qualitative component did not capture the provider perspectives on telemonitoring services in GDM management, which could have offered valuable insights.

## Conclusion

This study concludes that nurse-led telemonitoring is a feasible and well-accepted tool to facilitate close monitoring of pregnant women with GDM in a peri-urban hospital setting in Nepal. Additional large-scale studies are necessary to comprehensively assess its economic implications and effectiveness in improving clinical outcomes in women with GDM.

## Data Availability

Data will be available on reasonable request to the author.
